# Adherence to direct oral anticoagulant treatment for atrial fibrillation in the Netherlands: A surveillance study

**DOI:** 10.1002/pds.5242

**Published:** 2021-05-04

**Authors:** Gilda D. Zielinski, Nienke van Rein, Martina Teichert, Frederikus A. Klok, Frits R. Rosendaal, Felix J. M. van der Meer, Menno V. Huisman, Suzanne C. Cannegieter, Willem M. Lijfering

**Affiliations:** ^1^ Department of Clinical Epidemiology Leiden University Medical Center Leiden The Netherlands; ^2^ Department of Clinical Pharmacy & Toxicology Leiden University Medical Center Leiden The Netherlands; ^3^ Department of Thrombosis and Haemostasis Leiden University Medical Center Leiden The Netherlands

**Keywords:** adherence, anticoagulants, atrial fibrillation, discontinuation, pharmacoepidemiology, risk

## Abstract

**Background:**

Adherence to direct oral anticoagulants (DOACs) in patients with atrial fibrillation in every day practice may be less than in clinical trials.

**Aims:**

To assess adherence to DOACs in atrial fibrillation patients in every day practice and identify predictors for non‐adherence.

**Methods:**

Individual linked dispensing data of atrial fibrillation patients who used DOACs were obtained from the Foundation for Pharmaceutical Statistics covering the Netherlands between 2012 and 2016. One year adherence to DOAC was calculated for initial DOAC as proportion of days covered (PDC) ≥80% and the association between clinical variables and adherence was assessed using logistic regression. In addition, we measured non‐persistence, that is, patients who completely stopped their initial DOAC within 1 year follow‐up.

**Results:**

A total of 4797 apixaban‐, 20 454 rivaroxaban‐ and 18 477 dabigatran users were included. The mean age was 69 years (n = 43 910), which was similar for the DOAC types. The overall proportion of patients with PDC ≥80% was 76%, which was highest for apixaban‐ (87%), followed by dabigatran‐ (80%) and rivaroxaban (69%) users. Multivariable analyses revealed that age ≤60 years, no concomitant drug use were predictors for non‐adherence. Of atrial fibrillation patients who continued treatment, 97% had a PDC ≥80%, compared with only 56% for those who discontinued their DOAC treatment within 1 year.

**Conclusions:**

Non‐adherence to DOACs was associated with age ≤60 years and no concomitant drugs use. Non‐adherence was higher in patients who later discontinued DOAC treatment. Results of our study support research into interventions to improve adherence.

**Key Points**

- An advantage of direct oral anticoagulants (DOACs) over vitamin K antagonists (VKAs) is that they do not require regular monitoring but this may lead to poorer adherence.
- Non‐adherence was associated with younger age and no use of concomitant drugs.
- Adherence was higher for patients who continued their treatment, compared with those who discontinued DOAC treatment.
- Early (within 6 months) non‐adherence to DOAC was associated with highest non‐persistence rates.

## 1 INTRODUCTION

Guidelines of prevention of thromboembolism in patients with atrial fibrillation advocate direct oral anticoagulants (DOACs) as first line treatment.^1^ However, although a potential advantage of the DOACs over the treatment with vitamin K antagonists (VKAs) is that they do not require regular monitoring, non‐monitoring may lead to poorer drug adherence.^2^ Good adherence of over 95% to DOACs was reported in the DOAC clinical trials,^3^ but the patients in these studies were closely followed, and it is recognized that such levels of adherence are not seen in clinical practice. Indeed, observational studies have shown poorer adherence rates to DOAC than reported in clinical trials, with 60%–75% being fully adherent over a 12–24 months follow‐up period.^4^ This is problematic as studies have shown that non‐adherence to DOAC is associated with an increased risk of all‐cause mortality and thromboembolic stroke.^5^, ^6^, ^7^, ^8^, ^9^ Prior studies with both VKAs as DOACs have shown that adherence can be improved by frequent patient contacts and identifying non‐adherent patients through involvement of pharmacies and/or anticoagulation clinics.^10^, ^11^, ^12^ Moreover, age, sex, previous VKA use, high or low DOAC dose are associated with poor adherence, as these predictors have been shown to be associated with poor drug adherence in previous studies.^13^, ^14^

The aim of our study was to assess the 1 year adherence to DOAC (i.e., apixaban, rivaroxaban or dabigatran) in a large population of patients with atrial fibrillation. In addition, we evaluated whether demographic and socioeconomical characteristics like age, sex, and socioeconomic status were related with better adherence and whether early non‐adherence to DOAC led to non‐persistence.

## 2 METHODS

### 2.1 Study population

#### 2.1.1 Setting and databases

Patterns of drug use can be studied from pharmacy dispensing information.^15^ In the Netherlands, the Foundation for Pharmaceutical Statistics (SFK) gathers pharmacy dispensing data from >95% of community pharmacies but does not contain information on clinical indication or outcome.^16^ SFK data provide detailed information on the drugs dispensed, including the codes from the Anatomic–Therapeutic–Chemical (ATC) system of the World Health Organization,^17^ the prescribed dose, and the amount dispensed. In the current study, we collected data on DOAC use (by ATC code), with the DOAC dose, number of tablets dispensed, date of dispensing, patient's sex, age, any concomitant medical therapy, and if a patient used VKA prior to DOAC initiation or switched to VKA during follow‐up. Four digit postcodes of the patients were also provided by SFK which allowed us to characterize neighborhood socioeconomic status. The latter information was retrieved by using information from the Netherlands Institute of Social Research, which keeps record of neighborhood socioeconomic status by use of four‐digit postcodes.^18^ Data of patients and pharmacies were received anonymized. No patients were involved in setting the research question or the outcome measures, nor were they involved in developing plans for design or implementation of the study.

### 2.2 Inclusion criteria

We included all patients who had at least one delivery of the DOAC agents dabigatran and rivaroxaban from January 1, 2012 until April 1, 2016.

The index date for dabigatran and rivaroxaban users was the first dispensing between April 1, 2012 and April 1, 2015, To include only incident users and those who could have at least 1 year of follow‐up.

The index date for apixaban users was the first dispensing between April 1, 2013 and April 1, 2015, as it was registered in the Netherlands in April 2013 for the prevention of systemic embolism in atrial fibrillation. To include only incident apixaban users and those who could have at least 1 year of follow‐up. Therefore, the final inclusion period ran between April 1, 2012 and April 1, 2015, and all included patients were followed for a maximum of 1 year. The DOAC edoxaban was not included since it was not yet approved in the Netherlands during the time period studied.

### 2.3 Exclusion criteria

To maximize the chance that only incident DOAC users were included, we excluded patients who had received dabigatran or rivaroxaban January 1, 2012 and April 1, 2012. DOAC users who initiated treatment after April 1, 2015 were excluded as they could not be followed for 1 year Although SFK does not dispose the clinical indications for the drugs dispensed, the DOAC indication could be assessed on the basis of the first dose of DOAC, which is different for short term thromboprophylaxis, venous thrombosis treatment and thromboembolic prevention in atrial fibrillation patients.^19^ In the current study, only patients using a dosage corresponding to atrial fibrillation were included. Of note, patients who used apixaban 2.5 mg twice daily (bid) for less than 6 weeks could have done so both for venous thrombosis prophylaxis or for thromboembolic prevention in atrial fibrillation. Since we could not distinguish between these two possibilities, these patients were excluded from further analysis (n = 11 apixaban users, who initiated use before April 1, 2015).

### 2.4 Definition of follow‐up

Patients were followed in the SFK database starting from their first prescription at some point between April 1, 2012 until April 1, 2015. Follow‐up ended at 1 year of DOAC use or when they were considered non‐persistent.

### 2.5 Definition of adherence

Patient use of a DOAC was considered from the day of filling the prescription. The end of that prescription period was defined as the number of capsules/tablets filled divided by the prescribed numbers of capsules/tablet per day. Patients were considered non‐persistent if they did not file a new prescription within 90 days after ending the previous one.

Adherence to DOAC was defined as a dichotomous variable for a proportion of days covered (PDC) of at least 80%. This PDC cut‐off is consistent with published research.^5^, ^20^, ^21^ The PDC was calculated between the timeframe of the first DOAC prescription of initial DOAC treatment to the end of follow‐up. Since patients may stockpile their medications at home, overlaps between prescription were allowed and were included in the calculation of the PDC. Adherence levels substantially higher than 80% are required to prevent conditions in which the missing of even one pill can influence disease outcome, as for example is the case for anti‐retroviral therapy in HIV disease or oral contraceptive use to prevent pregnancy.^21^, ^22^ This is also true for DOAC use since its half‐life is short (9–17 h), and anticoagulation levels can return to normal even when missing one daily dose.^23^, ^24^, ^25^ Therefore, we also calculated the PDC of at least 95% and of at least 99%. As several studies have shown that a 90% adherence to DAOC results in better clinical outcomes, we added a PDC of 90%.^7^, ^8^

### 2.6 Definition of discontinuers

Patients who did not fill a new dispensing within 6 months of the previous one, were considered to have discontinued their initial DOAC treatment (that is stopped for >90 days or switched to another oral anti‐coagulant).^26^

### 2.7 Exposure variables

Patients were classified as dabigatran, rivaroxaban or apixaban users if they received at least one dispensing of ATC codes B01AE07, B01AF01 or B01AF02, respectively. DOACs can be administered to patients with atrial fibrillation in different dosages.^19^ For this purpose we classified dabigatran users as high dose users when they received a first dabigatran prescription of 150 mg bid. and as low dose users when they received 110 mg bid as a first dabigatran prescription. For rivaroxaban, a high dose user was defined as taking rivaroxaban 20 mg once daily (od), and a low dose user when taking rivaroxaban 15 mg od. For apixaban, a high dose user was defined when taking apixaban 5 mg twice daily (bid), and a low dose user when taking apixaban 2.5 mg bid. As described above, patients who were classified as having atrial fibrillation in the current study had to receive apixaban 2.5 mg bid dosage for more than 6 weeks.

### 2.8 Concomitant variables

If patients had received VKA (ATC code B01AA) or any other concomitant medication within 180 days prior to baseline, we defined them as previous VKA user or concomitant drug user, respectively. SFK did not give us any information on which concomitant medication was used by the DOAC users, and we could not distinguish if patients used the same medication over time or different medications. We therefore decided to the information we did have as a dichotomous variable.

Neighborhood socioeconomic status was gathered by using the database “status score” from the Netherlands Institute for Social Research.^18^ The “status score” of a neighborhood (postal code area) is based on (1) mean household income, (2) the percentage of households with a low income, (3) the percentage of inhabitants without a paid job and (4) the percentage of households with on average a low education. The status score combines these four variables into a continuous variable where the higher the score, the higher the socioeconomic status of a neighborhood is. We a‐priori defined a high neighborhood socioeconomic status as >90th percentile of status score in the SFK data.

### 2.9 Statistical analysis

Baseline characteristics of the DOAC users are expressed as numbers and percentages, or as means and standard deviations (SD).

Adherence rates were calculated as described earlier, and PDC levels of ≥80%, ≥90%, ≥95%, ≥99% are shown for all DOACS together, and were stratified for each DOAC type. Subsequently, we stratified the this analysis for continuers and discontinuers during the 1 year of follow‐up and the mean PDC's were calculated for the patients with a mean PDC ≥80% and <80%. This yielded the following groups; that is, all patients included, mean PDC of all DOAC users with a PDC <80% and a PDC ≥80%, continuers, mean PDC of all DOAC users with a PDC <80% and a PDC ≥80%, discontinuers mean PDC of all DOAC users with a PDC <80% and a PDC ≥80%.

Available patient characteristics (age, sex, previous VKA use, high or low DOAC dose, socioeconomic status and concomitant drug use) were included in the multivariable logistic regression analysis with a stepwise procedure to identify predictors that could be associated with poor adherence, as these predictors have been shown to be associated with poor drug adherence in previous studies.^13^, ^14^

From the SFK database we cannot rule out the possibility that a patient retrieved medication from different pharmacies at different times. If this occurs, it would seem that a patient was non‐adherent in pharmacy A, while the drugs were retrieved first in pharmacy A and then in pharmacy B. To account for such possible overestimation of non‐adherence, we excluded (in a sensitivity analysis) all patients who had the same birth year, sex, postal code, concomitant drug use, previous VKA use and who received the same initial DOAC during the observation period as another patient in the register, and repeated the aforementioned analysis to see if this would influence the main results.

All statistical analyses were performed with SPSS for Windows, release 24.0 (SPSS. Chicago, IL).

## 3 RESULTS

### 3.1 Study population

**FIGURE 1:**
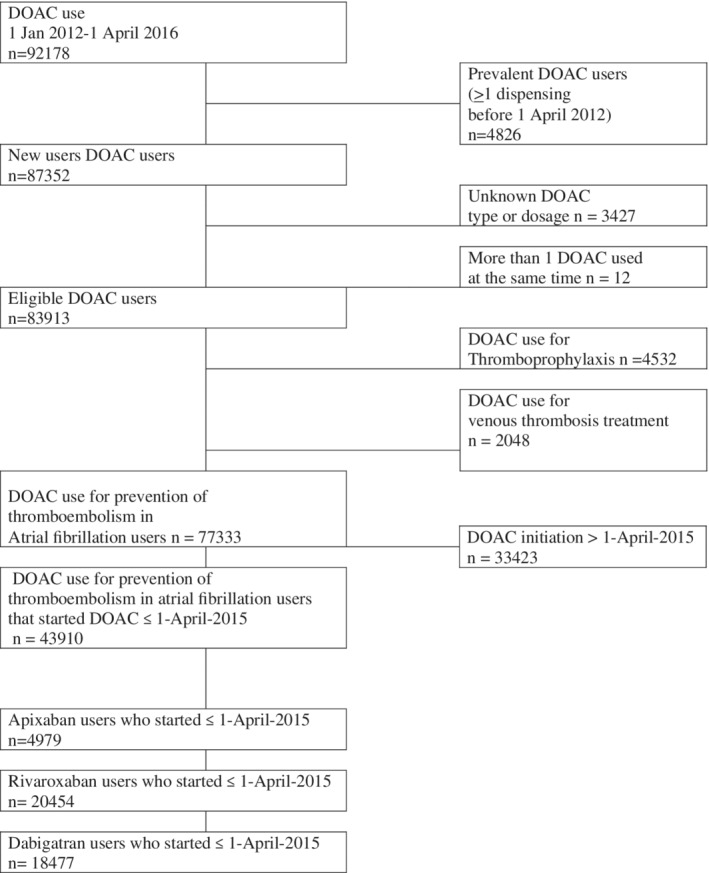
Flow chart

We identified 92 718 patients who initiated treatment with DOAC between January 1, 2012 and April 1, 2016, based on the data provided by 1538 pharmacies in the Netherlands (79% of the total number of 1981 community pharmacies in the Netherlands in 2015).^16^ Figure 1 describes the process of sample selection. After we applied the inclusion criteria in which we separated prevalent DOAC users (n = 4826) from incident DOAC users (n = 87 352), and excluded patients in whom the DOAC type or dosage (n = 3427) was not reported or in whom two or more DOACs were prescribed at the same time (n = 12), there were 83 913 DOAC users of whom the majority (n = 77 333) were identified as using a dosage in accordance with atrial fibrillation. There were n = 43 910 who initiated their DOAC before April 1, 2015 (as these patients could be followed at least 1 year). Baseline characteristics of these DOAC patients are shown in Table 1. The mean ages of the patients using apixaban (70 years; SD 10 years), dabigatran (70 years; SD 10 years) or rivaroxaban (69 years; SD 11 years) were similar. Most patients used rivaroxaban (n = 20 454; 47%), followed by dabigatran (n = 18 477; 42%) and apixaban (n = 4979; 11%). The large majority (≥99%) of patients on DOACs had not used a VKA before DOAC initiation, which follows clinical guidelines in the Netherlands that recommend DOAC over VKA as anticoagulant treatment since 2016.^27^

**TABLE 1:** Baseline characteristics of the atrial fibrillation patients

	DOAC		Apixaban		Rivaroxaban		Dabigatran	
*Any dose*, *n*	*43 910*		*4979*		*20 454*		*18 477*	
Mean age, years (SD)	69	(10)	70	(10)	69	(11)	70	(10)
Men, n (%)	23 691	(54)	2832	(57)	10 290	(50)	10 569	(57)
Concomitant drug use, n (%)	38 631	(88)	4545	(91)	17 667	(86)	16 419	(89)
Previous exposure to VKA, n (%)	33	(0.1)	14	(0.3)	10	(0)	9	(0)
Socioeconomic class^a^								
>90% percentile, n (%)	4250	(10)	407	(8)	2007	(10)	1836	(10)
*Low dose*, *N*	*9198*		*739*		*2047*		*6412*	
Mean age, years (SD)	74	(10)	80	(9)	75	(10)	73	(10)
Men, n (%)	4486	(49)	309	(42)	995	(49)	3182	(50)
Concomitant drug use, n (%)	8175	(89)	689	(93)	1861	(91)	5625	(88)
Previous exposure to VKA, n (%)	6	(0.1)	0	(0)	3	(0)	3	(0)
Socioeconomic class^a^								
>90% percentile, n (%)	902	(10)	55	(8)	226	(11)	621	(10)
*High dose*, *N*	*34 712*		*4240*		*18 407*		*12 065*	
Mean age, years (SD)	68	(10)	69	(10)	68	(11)	68	(10)
Men, n (%)	19 205	(55)	2523	(60)	9295	(51)	7387	(61)
Concomitant drug use, n (%)	30 456	(88)	3856	(91)	15 806	(86)	10 794	(90)
Previous exposure to VKA, n (%)	27	(0.1)	14	(0.3)	7	(0)	6	(0)
Socioeconomic class^a^								
>90% percentile, n (%)	3348	(10)	352	(8)	1781	(8)	1215	(10)

^a^ According to Statusscore of the Sociaal en Cultureel Plan Bureau, the Netherlands.

### 3.2 Non‐adherence, overall findings

The overall proportion of patients with PDC ≥80% was 76%. A PDC ≥80% was most often found in apixaban users (87%), and least found for rivaroxaban users (69%; Table 2). As expected, the percentages of patients who adhered to their medication during follow‐up declined if a more stringent definition of adherence was used (Table 2). If a PDC of 95% is used, 65% of the apixaban‐, 55% of the dabigatran‐ and 44% of the rivaroxaban users would have appropriate adherence. For a PDC ≥99% the adherence rate was 37% for apixaban‐, 32% for dabigatran‐ and 23% for rivaroxaban users.

**TABLE 2:** Adherence to DOAC in patients with atrial fibrillation who use a DOAC for thromboembolic prevention

	DOAC (n = 43 910)		Apixaban (n = 4979)		Rivaroxaban (n = 20 454)		Dabigatran (n = 18 477)	
PDC ≥80%, n (%)	33 165	(76)	4347	(87)	14 070	(69)	14 748	(80)
PDC ≥90%, n (%)	26 603	(61)	3753	(75)	10 830	(53)	1202	(65)
PDC ≥95%, n (%)	22 388	(51)	3235	(65)	9072	(44)	10 081	(55)
PDC ≥99%, n (%)	12 466	(28)	1826	(37)	4794	(23)	5846	(32)
Sensitivity analysis^a^	(n = 29 881)		(n = 3978)		(n = 13 495)		(n = 12 408)	
(PDC ≥80%, (n %)	22 314	(75)	3444	(87)	9111	(68)	9759	(79)

Abbreviations: PDC, proportion of days covered; VKA, vitamin K antagonist.

^a^ Patients with the same birth year, sex, concomitant drug use, previous VKA use and who received the same initial DOAC, excluded to avoid potential duplicates.

### 3.3 Variables related with non‐adherence in DOAC users

In multivariable analysis, adherence was related to concomitant drug use, where those who used concomitant drugs better adhered to DOAC (Table 3), as did elderly patients. Other variables, including sex, previous VKA use, DOAC dose and socioeconomic status showed no consistent associations with adherence for the DOACs tested.

**TABLE 3:** Adherence (PDC ≥80%) to DOAC in patients with atrial fibrillation: Subgroup analysis

	PDC ≥80%. No./Total No. (%)	Odds ratio (95% CI)	Odds ratio (95% CI)^a^
*Apixaban*			
VKA naïve	4334/4965 (87)	1 reference	1 reference
VKA experienced	13/14 (93)	1.89 (0.25–14.5)	1.62 (0.21–12.5)
High dose DOAC	3704/4240 (87)	1 reference	1 reference
Low dose DOAC	643/739 (87)	0.97 (0.77–1.22)	0.79 (0.61–1.02)
Age ≤60 years	579/699 (82)	1 reference	1 reference
Age 60–75 years	2304/2636 (87)	1.44 (1.15–1.81)	1.38 (1.09–1.75)
Age >75 years	1464/1644 (89)	1.69 (1.31–2.17)	1.62 (1.23–2.13)
Men	2448/2832 (86)	1 reference	1 reference
Women	1899/2147 (88)	1.20 (1.01–1.42)	1.13 (0.94–1.35)
No concomitant drug	271/434 (62)	1 reference	1 reference
Concomitant drug use	4076/4545 (90)	5.23 (4.21–6.49)	5.18 (4.16–6.45)
Socioeconomic class^b^			
<25th perc, n (%)	1160/1332 (87)	1 reference	1 reference
25–75th perc, n (%)	2315/2636 (88)	1.07 (0.88–1.30)	1.08 (0.88–1.33)
75–90th perc, n (%)	499/576 (87)	0.96 (0.72–1.28)	0.99 (0.74–1.34)
>90% perc, n (%)	349/407 (86)	0.89 (0.65–1.23)	0.91 (0.66–1.27)
*Rivaroxaban*			
VKA naïve	14 061/20 444 (69)	1 reference	1 reference
VKA experienced	9/10 (90)	4.09 (0.52–32.25)	2.73 (0.34–21.74)
High dose DOAC	12 369/18 407 (67)	1 reference	1 reference
Low dose DOAC	1701/2047 (83)	2.40 (2.13–2.71)	2.34 (2.06–2.64)
Age ≤60 years	2367/3640 (65)	1 reference	1 reference
Age 60–75 years	7629/11 053 (69)	1.85 (1.10–1.28)	1.19 (1.10–1.29)
Age >75 years	4065/5761 (71)	1.28 (1.17–1.39)	1.20 (1.10–1.32)
Men	7734/10 290 (75)	1 reference	1 reference
Women	6336/10 164 (62)	0.55 (0.52–0.58)	0.53 (0.50–0.56)
No concomitant drug	1503/2787 (54)	1 reference	1 reference
Concomitant drug use	12 567/17 667 (71)	2.11 (1.94–2.28)	2.10 (1.93–2.28)
Socioeconomic class^b^			
<25th perc, n (%)	3430/4922 (70)	1 reference	1 reference
25–75th perc, n (%)	6833/10 306 (66)	0.86 (0.80–0.92)	0.86 (0.80–0.93)
75–90th perc, n (%)	2206/3095 (71)	1.08 (0.98–1.19)	1.08 (0.98–1.12)
>90% perc, n (%)	1513/2007 (75)	1.33 (1.18–1.50)	1.30 (1.15–1.47)
*Dabigatran*			
VKA naïve	14 743/18 468 (80)	1 reference	1 reference
VKA experienced	5/9 (56)	0.32 (0.09–1.18)	0.28 (0.07–1.03)
High dose DOAC	9902/12 065 (82)	1 reference	1 reference
Low dose DOAC	4846/6412 (76)	0.68 (0.63–0.73)	0.65 (0.60–0.71)
Age ≤60 years	2148/2934 (73)	1 reference	1 reference
Age 60–75 years	8071/9974 (81)	1.55 (1.41–1.71)	1.60 (1.45–1.76)
Age >75 years	4529/5569 (81)	1.59 (1.43–1.77)	1.91 (1.70–2.13)
Men	8715/10 569 (83)	1 reference	1 reference
Women	6033/7908 (76)	0.69 (0.64–0.74)	0.66 (0.62–0.72)
No concomitant drug	1367/2058 (66)	1 reference	1 reference
Concomitant drug use	13 381/16 419 (82)	2.23 (2.02–2.46)	2.17 (1.96–2.40)
Socioeconomic class^b^			
<25th perc, n (%)	3474/4301 (81)	1 reference	1 reference
25–75th perc, n (%)	7457/9297 (80)	0.97 (0.88–1.06)	0.95 (0.87–1.05)
75–90th perc, n (%)	2294/2951 (78)	0.83 (0.74–0.93)	0.81 (0.72–0.91)
>90% perc, n (%)	1453/1863 (79)	0.90 (0.79–1.03)	0.90 (0.79–1.04)

Abbreviations: PDC, proportion of days covered; perc, percentile; VKA, vitamin K antagonist.

^a^ Multivariable adjusted.

^b^ According to Statusscore of the Sociaal en Cultureel Plan Bureau, the Netherlands.

**FIGURE 2:**
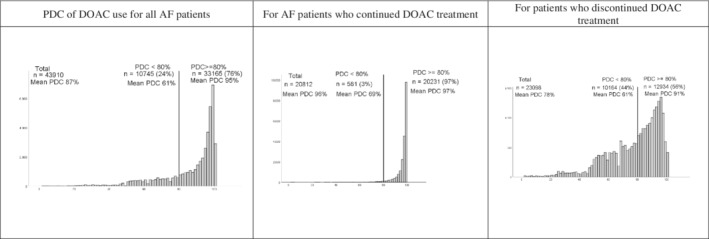
Mean PDC, % of AF patients with a PDC <80% and PDC ≥80% for DOAC use; stratified for continuance and discontinuance. AF, atrial fibrillation; DOAC, direct oral anticoagulants; PDC, proportion of days covered

**FIGURE 3:**
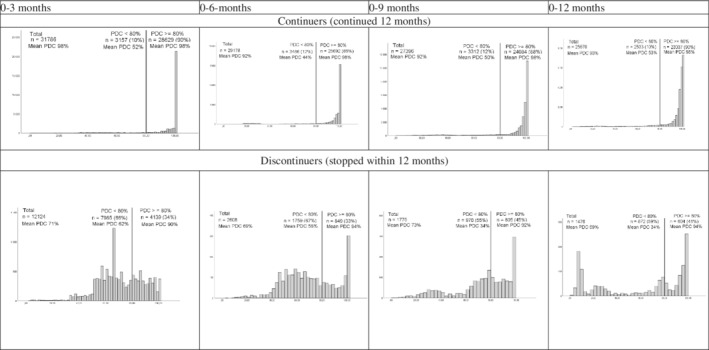
Mean PDC, % of AF patients with a PDC <80% and PDC ≥80% for DOAC use; stratified for continuance and discontinuance at 3, 6, 9 and 12 months. AF, atrial fibrillation; DOAC, direct oral anticoagulants; PDC, proportion of days covered

Next, we stratified adherence to DOAC for (dis)continuation with DOAC, to evaluate if patients who discontinued their treatment adhered differently (Figures 2 and 3). We found that patients who discontinued their DOAC treatment had lower adherence rates, throughout the 1 year follow‐up. Among patients who discontinued their treatment, 56% had a PDC of ≥80%, compared to 97% for the patients who continued their treatment. Adherence was also the lowest for the patients who discontinued their treatment in the first 3–12 months of follow‐up (34% at 3 months, 33% at 6 months, 45% at 9 and 41% at 12 months; Figure 3).

## 4 DISCUSSION

In this population based study in patients using a DOAC dosage in accordance with atrial fibrillation, we observed that 76% of the patients adhered to DOAC when the PDC was set at ≥80%. Multivariable analyses revealed that younger age and no concomitant drug use were predictors for non‐adherence. We also observed that the proportion of patients using a DOAC with a PDC of ≥80% in our study was lower for those who discontinued treatment (56%, compared with 97% of the continuers), and lowest for the patients who discontinued their treatment in the first 3–6 months of follow‐up (34% at 3 months, 33% at 6 months, 45% at 9 months and 41% at 12 months). Of the DOACs that were tested, apixaban had the best adherence profile (87% users had a PDC of ≥80%, compared with 80% of the dabigatran users and 69% of the rivaroxaban users). In the current study, few patients switched from VKA to DOAC (<1%). This is most likely related with the timeframe that our study was conducted (April 1, 2012 until April 1, 2015). According to the Dutch guidelines at that time patients using VKA were not switched to a DOAC.^27^, ^28^, ^29^ Switching from VKA to a DOAC commenced in 2016, and has increased since that time.^20^, ^29^ Therefore these patients were not available for inclusion during our study period. This is also confirmed in our previous study using the same data base, but also included the patients that started using DOAC in 2016. Previous VKA use in this population was 8%.^26^

### 4.1 Comparison with other studies

We found 11 observational studies^4^, ^5^, ^13^, ^14^, ^30^, ^31^, ^32^, ^33^, ^34^, ^35^, ^36^ that investigated adherence of patients to DOAC treatment of which 7 were restricted to atrial fibrillation patients with a follow‐up of more than 6 months.^4^, ^5^, ^14^, ^30^, ^31^, ^32^, ^33^ The proportion of atrial fibrillation patients with a PDC of ≥80% in five of these studies ranged from 72% to 77%,^4^, ^5^, ^14^, ^30^, ^31^, ^32^, ^33^ which is similar to what we found in our study. The study by Maura et al., which included a total of 22 267 patients with atrial fibrillation, using either dabigatran (n = 11 141) or rivaroxaban (n = 11 126) found a relatively low proportion of patients using a DOAC with a PDC of ≥80% (61%). However after excluding switchers and patients who died, the proportion of patients using a DOAC with a PDC of ≥80% increased to a comparable 71%.^32^ In a study by Coleman et al., in which atrial fibrillation patients using either rivaroxaban (n = 11 052) or dabigatran (n = 11 100) were included.^31^ the proportion of patients using rivaroxaban with a PDC≥80% was 74% at 6 months and 62% at 12 months of follow‐up and 65% at 6 months and 52% at 12 months for dabigatran. This adherence rate is lower as compared with our study. The difference might be due to the definition of follow‐up that was used (at 12 months 40%–65% of patients on rivaroxaban and dabigatran were still followed, while in our study all patients were followed for 12 months). The relatively low proportion of patients with a PDC ≥80% during early follow‐up is in line with an observational study of Yao et al., in which atrial fibrillation patients were included initiating either rivaroxaban (n = 12 336), dabigatran (n = 10 235) or apixaban (n = 3900; 47.5%) with a maximum follow‐up time of 6 months. The proportion of patients with a PDC of ≥80% was highest for apixaban (62%), compared to the dabigatran (39%) and rivaroxaban (51%) users.^36^ The other four observational studies that investigated adherence of patients to their DOAC treatment included patients using an unspecified DOAC.^13^, ^34^, ^35^, ^36^ The proportion of atrial fibrillation patients with a PDC ≥80% ranged from 67% up to 88%.^13^, ^34^, ^35^, ^36^ The wide range can be explained by differences in follow‐up time, patients included, differences in PDC definitions and DOAC type. The proportion of atrial fibrillation patients with a PDC ≥80% was lowest (67%) in a study by Tsai et al., in which atrial fibrillation patients with warfarin experience (n = 10 369) and without warfarin experience (n = 7322) were included in a study using pharmacy claims database, with a maximum follow‐up of 6 months.^37^ In a study of van den Heuvel et al., the highest proportion of patients with a PDC of ≥80% (88%) was found, but that study excluded patients with only one DOAC prescription and the period with a last valid dispensing was disregarded which could explain the high adherence rate.^38^ In a study by Harper et al., there were 43 339 patients with atrial fibrillation on dabigatran followed for 1 year.^14^ Similar to our results, they found the lowest adherence rate among the youngest patients, which was is also shown in a study by Perreault et al.^39^ To summarize, most of these aforementioned studies show a relatively high proportion of atrial fibrillation patients on DOAC with a PDC around the accepted adherence of ≥80%.^4^, ^5^, ^30^, ^31^, ^38^, ^39^, ^40^, ^41^

The main concern with poor adherence to anticoagulants is the risk of thromboembolism. Ideally for the greatest benefit treatment should be continuous which certainly applies to DOACs as these drugs have a short half‐life and a break in treatment can rapidly decrease their efficacy.^5^, ^24^, ^42^, ^43^ Based on the assumption that a higher PDC is less likely to put a patient at risk, we found that the adherence for a more stringent PDC of 95% was 66% for apixaban, 56% for dabigatran and 51% for rivaroxaban. It was even less for an ideal PDC of ≥99% (45% for apixaban, 36% for dabigatran and 32% for rivaroxaban). These numbers raise concerns that the reduction in the risk of stroke expected from DOACs may not be optimal in every day practice due to a low proportion of patients who achieve a PDC of 95% or higher. Potentially the adherence rate to DOAC could be improved by for instance specific pharmacist‐based activities, such as telephone contact or face to face contact when a patient is non‐adherent to DOAC.^44^

### 4.2 Strengths and weaknesses of this study

Strengths of our study are its population‐based design and unselected participants. A limitation of this study is that SFK does not dispose information on the exact indication for DOAC treatment, although we could approximate this by the difference in first dose of DOAC for atrial fibrillation as compared with venous thrombosis or thromboprophylaxis. Other population based studies where the actual diagnosis of atrial fibrillation was known^4^, ^5^, ^32^ shows similar non‐adherence (and non‐persistence) rates to DOAC over a 12 month follow‐up time making it likely that our used strategy in determining who had atrial fibrillation in the SFK database is valid. Another potential limitation is that SFK was only able to provide data of 79% of all pharmacies in the Netherlands. Reasons are that not all pharmacies had provided complete data during the study period without switches in software systems. Therefore, reasons for not including these pharmacies were considered completely at random and are not expected to influence our results. Another limitation of this study is that we do not know the reasons why patients did not adhere or discontinued their anticoagulant treatment as this information was not available in our data sources. However, it seems unlikely that cardioversion or return to sinus rhythm (after which a patient is allowed to stop anticoagulation) can fully explain the high non‐adherence and discontinuation rate to DOAC that we found in our study as only a minority of patients with atrial fibrillation return to sinus rhythm after cardioversion (6%–9% within 1.5 years after atrial fibrillation onset).^45^, ^46^ A further limitation is that SFK could only provide us with few characteristics that we could relate with non‐adherence and that the precise use of concomitant medications was not available. A final limitation is that we had access to dispensing data only and that we had to make assumptions on when a patient became non‐adherent or non‐persistent. However, the assumptions we used are widely used in other pharmacoepidemiologic studies on adherence and persistence.^21^

In conclusion, non‐adherence to DOACs was associated with age ≤60 years and no concomitant drugs use. Non‐adherence was higher in patients who later discontinued DOAC treatment. Results of our study support research into interventions to improve adherence.

## CONFLICT OF INTEREST

The manuscript has been read and approved for submission by all authors. The work described in this manuscript is original and has not been previously published or presented. G.D.Z., N.V.R., W.M.L., M.T., F.A.K., F.J.M.V.D.M., F.R.R. and S.C.C. have no conflict of interest to declare. M.V.H. reports receiving grant support from Boehringer Ingelheim, GlaxoSmithKline, and Aspen, and lecture fees from Bristol‐Myers Squibb/Pfizer, Boehringer Ingelheim, and Bayer HealthCare.

## AUTHOR CONTRIBUTIONS

Gilda D. Zielinski: analysis and interpretation of data, drafting of the manuscript. Martina Teichert: technical support, critical revision of the manuscript for important intellectual content. Nienke van Rein: critical revision of the manuscript for important intellectual content. Frederikus A. Klok: critical revision of the manuscript for important intellectual content. Felix J. M. van der Meer: critical revision of the manuscript for important intellectual content. Frits R. Rosendaal: critical revision of the manuscript for important intellectual content. Menno V. Huisman: critical revision of the manuscript for important intellectual content. Suzanne C. Cannegieter: study supervision, critical revision of the manuscript for important intellectual content. Willem M. Lijfering: Acquisition of data, study concept and design, supervision of analysis and interpretation of data.

## ETHICS STATEMENT

Data of patients and pharmacies were coded and anonymized prior to analysis. Use of anonymous administrative data in descriptive studies in the Netherlands is not considered as an interventional study according to Dutch legislation, and therefore does not need to be submitted to a medical ethic committee for approval.
